# Supplementary material for: Blood-Based Immune Profiling Combined with Machine Learning Discriminates Psoriatic Arthritis from Psoriasis Patients
Source: Int J Mol Sci. 2021 Oct 12;22(20):10990. doi: 10.3390/ijms222010990 (PMC8538368; doi:10.3390/ijms222010990)
Supplement: Supplementary file 1 [file ijms-22-10990-s001.zip › ijms-1408687-supplementary.pdf]

SUPPLEMENTARY TABLES

Supplementary Table S1: FACS staining panels used in this study.

	**FITC /AF488**	**PE**	**ECD**	**PerCp5.5**	**PC7**	**APC /AF647**	**APC-AF700**	**APC-AF750 /APC-Cy7**	**e450 /BV421**	**Krome Orange**
**Panel 1 (LMI1)**	CD16 (clone 3G8)	HLA-DR (clone immu-357)	CD14 (clone RMO52)	CD4 (clone 13B8.2)	CD25 (clone M-A251)	CD56 (clone N901)	CD8 (clone B9.11)	CD19 (clone J3-119)	CD3 (clone UCHT1)	CD45 (clone J33)
**Panel 2 (LMI2)**	CD45RA (clone ALB11)	CD3 (clone UCHT1)	CD45RO (clone UCHL1)	CD27 (clone 1A4CD27)	CD25 (clone M-A251)	CD56 (clone N901)	CD127 (clone R34.34)	CD8 (clone B9.11)	CD4 (clone 13B8.2)	CD45 (clone J33)
**Panel 3 (LMI3)**	IgD (clone IADB6)	IgM (clone SA-DA4)	CD3 (clone UCHT1)	CD27 (clone 1A4CD27)	CD38 (clone LS198-4-3)	CD24 (clone ALB9)	CD5 (clone BL1a)	CD19 (clone J3-119)	CD20 (clone B9E9)	CD45 (clone J33)
**Panel 4 (LMI4)**	KI67 (clone B56)	HLA-DR (clone immu-357)	CD45RA (clone 2H4LDH11LDB9	CD4 (clone 13B8.2)	CD25 (clone M-A251)	Helios (clone 22F6)	CD127 (clone R34.34)	CD8 (clone B9.11)	FoxP3 (clone PCH101)	CD45 (clone J33)
**Panel 5 (LMI6)**	CD45RA (clone ALB11)	CD196 (clone 11A9)	CD8 (clone SFCI21Thy2D3)	CD183 (clone G025H7)	CD194 (clone 1G1)	CD25 (clone 2A3)	CD4 (clone RPA-T4)	CD195 (clone 2D7)	CD197 (clone G043H7)	CD45 (clone J33)

Supplementary Table S2: Univariate analysis of peripheral blood cell subsets in Pso vs PsA

Cell subsets	P-values	Log fold change of Pso vs PsA
CD196+Monocytes	1,78E-05	1,49
CD45RO+CD45RA-	3,57E-05	0,77
CD4+CD196+CD183-CD194+	2,06E-04	-0,62
CD4+CD197+CD45RA-	2,32E-04	0,62
CD8+CD45RO+CD27-	3,12E-04	1,44
CD8+CD45RA-CD27-	3,14E-04	1,47
CD4+CD25+CD127lo(Treg)	3,17E-04	0,46
CD4+CD45RO+CD27-	5,09E-04	0,89
CD4+CD45RA-CD25-CD196-CD183-CD194+	1,43E-03	-0,72
CD8+CD197+CD45RA+	1,55E-03	0,68
TregFOXP3+Helios+	1,87E-03	-0,09
Lymphocytes	2,01E-03	0,09
CD4+CD45RA-CD27-	2,51E-03	1,00
CD19+IgD+CD5++	2,54E-03	0,77
CD45RO-CD45RA+	2,66E-03	-0,12
CD4+CD25highTreg	3,51E-03	-0,27
CD4+CD196-CD183-CD194+	4,08E-03	-0,57
TregHLA-DR+	6,62E-03	0,49
CD8+CD196+CD183-CD194+	7,40E-03	-0,74
KI67+Treg	8,58E-03	-0,22
CD194+Monocytes	9,40E-03	0,29
CD4+CD197-CD45RA-	1,15E-02	-0,19
CD197+Monocytes	1,27E-02	1,36
CD8+CD196-CD183-CD194+	1,57E-02	-0,74
CD8+CD197-CD45RA+	1,78E-02	-0,27
CD4+	1,83E-02	0,01
Monocytes	2,09E-02	0,39
CD8+CD196+	2,11E-02	-0,39
CD8+CD196-	2,11E-02	0,21
CD4+CD45RA-CD25-CD196+CD183+CD194+	2,58E-02	-0,78
CD24+CD38+	3,22E-02	-0,01
CD4+CD45RA-CD25-CD196+CD183-CD194-	3,48E-02	0,32
CD4+CD45RA-CD25-CD196-CD183-CD194-	4,09E-02	0,27
CD14+CD16+	5,23E-02	-0,14
CD4+CD45RA-CD25-CD196+	5,42E-02	-0,12
CD4+CD45RA-CD25-CD196-	5,42E-02	0,23
CD27-IgM+CD24+CD38high	6,14E-02	0,45
IgM+CD38++CD27+	6,16E-02	0,62
CD4+CD45RA+CD27+	6,40E-02	-0,03
CD56+CD16-	6,98E-02	0,72

CD8+	7,64E-02	0,34
CD4+CD45RO-CD27-	9,43E-02	1,87
CD4+CD196-CD183-CD194-	9,55E-02	0,18
IgM-	9,61E-02	0,41
CD4+CD196-CD183+CD194-	1,14E-01	0,77
CD8+CD196-CD183-CD194-	1,15E-01	0,19
CD24++CD38++	1,16E-01	0,43
CD14++CD16-	1,32E-01	0,24
CD8+CD45RA+CD27+	1,45E-01	-0,02
IgD+CD5+	1,46E-01	0,07
CD4+CD197-CD45RA+	1,47E-01	-0,46
CD19+	1,48E-01	0,52
IgD+IgM+	1,66E-01	0,04
CD45+	1,66E-01	0,22
CD8+CD196+CD183+CD194-	1,83E-01	0,63
CD27-IgM+	1,89E-01	0,01
CD4+CD45RO-CD27+	1,99E-01	0,03
IgD-IgM-CD38++	2,13E-01	-0,27
IgD-IgM-CD27-	2,17E-01	0,33
CD19+CD20-	2,18E-01	0,54
CD19+CD20+	2,18E-01	0,12
CD4+CD45RA+CD27-	2,41E-01	1,33
CD195+Monocytes	2,42E-01	0,23
CD3-CD19+	2,58E-01	0,49
CD4+ CD8+ ratio	2,65E-01	-0,08
CD56+CD16+	2,76E-01	0,12
CD8+CD197-CD45RA-	2,81E-01	-0,03
IgD-IgM-	2,85E-01	0,32
KI67+CD8	3,04E-01	-0,20
IgD+IgM-	3,18E-01	0,36
CD4+CD8+	3,21E-01	1,28
CD4+CD196+CD183+CD194-	3,24E-01	0,45
CD4-CD8-	3,26E-01	0,24
CD8+CD45RO-CD27-	3,45E-01	-0,17
CD24+CD38+	3,49E-01	0,11
CD8+CD45RO-CD27+	3,50E-01	0,03
CD3-CD56+	3,57E-01	-0,06
CD8+CD197+CD45RA-	3,59E-01	0,35
TregCD25+CD127low	3,64E-01	0,30
IgD-CD5+	3,69E-01	0,29
KI67+CD4KI67+CD8ratio	3,70E-01	0,29
CD8+CD45RA+CD27-	4,14E-01	-0,13
CD4+CD45RA-CD27+	4,17E-01	0,22
nTreg	4,24E-01	-0,31
CD14++CD16+	4,37E-01	-0,06

LeukocytesCD45+	4,39E-01	0,17
IgD-CD5++	4,60E-01	-0,21
CD4CD25-CD127+	5,09E-01	0,11
CD4+CD45RA-CD25-CD196-CD183+CD194-	5,26E-01	0,43
NeutrophilsLymphocytesratio	5,30E-01	0,17
TregCD45RA-	5,36E-01	0,19
TregCD45RA+	5,36E-01	0,03
IgD+IgM+CD27-	5,74E-01	0,11
IgD+IgM+CD27+	5,74E-01	0,21
IgD-IgM+	5,78E-01	-0,07
IgM+CD27-	5,85E-01	0,11
CD8+CD45RA-CD27+	5,89E-01	0,21
CD8+CD196-CD183+CD194-	5,97E-01	0,34
KI67+CD4	6,02E-01	0,55
IgD-IgM-CD38+CD27+	6,55E-01	0,17
CD183+Monocytes	7,61E-01	0,04
CD4+CD197+CD45RA+	7,83E-01	0,16
IgD-IgM+CD27	7,90E-01	0,16
IgD-IgM+CD27-	7,92E-01	0,06
CD56++CD16-	8,01E-01	0,08
CD24+CD38+IgD+IgM+	8,24E-01	0,13
CD24+CD38+CD27+IgM+	8,26E-01	0,21
IgM+CD38+CD27+	8,28E-01	0,17
CD4+CD45RO+CD27+	8,90E-01	0,15
CD4+CD196-	8,91E-01	0,13
CD4+CD196+	8,91E-01	0,15
CD3+CD56+	9,03E-01	0,10
CD8+CD45RO+CD27+	9,38E-01	0,13
CD3+CD56-	9,43E-01	0,14
mTreg	9,44E-01	0,12
Neutrophils	9,59E-01	0,13
CD14+	9,83E-01	0,14
TregFOXP3+Helios-	9,94E-01	0,14
